# Identification of residues important for substrate uptake in a glucose transporter from the filamentous fungus *Trichoderma reesei*

**DOI:** 10.1038/srep13829

**Published:** 2015-09-08

**Authors:** Weixin Zhang, Yanli Cao, Jing Gong, Xiaoming Bao, Guanjun Chen, Weifeng Liu

**Affiliations:** 1 State Key Laboratory of Microbial Technology, School of Life Science, Shandong University, No.27 Shanda South Road, Jinan 250100, Shandong, P. R. China; 2 Cancer Research Center, School of Medicine, Shandong University, No.44 Wenhuaxi Road, Jinan 250012, Shandong, P. R. China

## Abstract

The glucose transporter is an important player in cell metabolism that mediates the intracellular uptake of glucose. Here, we characterized the glucose transporter Stp1 from the filamentous fungus *Trichoderma reesei*. The individual substitution of several conserved residues for Ala in Stp1 corresponding to those interacting with D-glucose in the xylose/H^+^ symporter XylE inflicted contrasting effects on its ability to support the growth of an *hxt*-null yeast on glucose. The targeted change of Phe 50, proximal to the substrate-binding site, was also found to exert a profound effect on the activity of Stp1. In contrast with the charged residues, the substitution of Phe 50 with either the hydrophilic residues Asn and Gln or the small residues Gly and Ala significantly enhanced the transport of glucose and its fluorescent analogue, 2-NBDG. On the other hand, a variant with the three substitutions I115F, F199I and P214L displayed remarkably improved activity on glucose and 2-NBDG transport. Further analysis indicated that the combined mutations of Ile 115 and Pro 214, positioned on the lateral surface of the Stp1 N-domain, fully accounted for the enhanced transport activity. These results provide insight into the structural basis for glucose uptake in fungal sugar transporters.

Glucose is the primary source of carbon and energy for most living organisms, from bacteria to humans. Glucose not only plays a vital role in cell metabolism and the maintenance of cellular homeostasis but also is a signalling molecule with various regulatory functions in gene transcription, enzyme activity, hormone secretion, etc^1^,^2^,^3^. The extracellular uptake of glucose across the plasma membrane, which is mediated by glucose transporters, represents a key step in glucose metabolism. Mammalian glucose transporters consist of glucose transport facilitators (GLUTs) and Na^+^-coupled glucose transporters (SGLT)^3^. In humans, 14 members of the GLUT family have been identified, with GLUT1 and GLUT4 being the best characterized^4^. The individual GLUT isoforms exhibit different substrate specificities, kinetic properties, and gene expression profiles and thus may play specific roles related to glucose handling to regulate metabolism, differentiation or oncogenesis^1^,^3^. In addition to mammalian GLUTs, glucose transporters have also been identified in eukaryotic microbes, including the baker’s yeast *Saccharomyces cerevisiae* and filamentous fungi. Twenty glucose (hexose) transporters (HXT1 to HXT17, GAL2, SNF3, and RGT2) have been identified in *S. cerevisiae*, among which SNF3 and RGT2 act as glucose sensors^5^. The different HXT proteins have been found to display distinct modes or kinetics of glucose uptake, although they are structurally similar^6^,^7^. In *Aspergillus niger*, *mstA* encodes a high-affinity sugar/H^+^ symporter that is regulated in response to extracellular pH^8^. In *Aspergillus nidulans*, the glucose transporter-encoding genes *hxtA*, *hxtB* (*mstC*), *hxtC*, *hxtD* (*mstA*), *hxtE* and *mstE* have been identified and preliminarily studied^9^,^10^,^11^,^12^. Despite considerable research on the prototype HXT proteins, the structural understanding of fungal glucose transporters is still limited.

GLUTs and their homologues belong to the major facilitator superfamily (MFS), an ancient superfamily that is the largest secondary transporter superfamily found in all kingdoms of life^13^, with over 10,000 members described within the Transporter Classification Database (TCDB, http://www.tcdb.org/)^14^. However, three-dimensional structures are only available for seventeen MFS proteins from ten subfamilies (http://blanco.biomol.uci.edu/mpstruc/). Despite weak sequence similarity and distinct substrate specificity, MFS members share a common structural fold known as the MFS fold. A canonical MFS fold comprises two halves, the amino- and carboxy-terminal domains, each containing six consecutive transmembrane helices. The proposed model for transport by MFS proteins is alternating access, in which the substrate-binding site is alternatively exposed to either side of the membrane^15^,^16^. More structural details relevant to glucose transport have been revealed by the resolved crystal structures of the xylose/H^+^ symporter XylE, a glucose transporter homolog from *Escherichia coli*, in a partially occluded outward-facing conformation bound to D-xylose or D-glucose^17^. The mechanism for glucose/H^+^ symport has also been proposed by mutagenesis and functional studies based on the crystal structure of the glucose transporter GlcP from *Staphylococcus epidermidis*^18^. Recently, the inward-open structure of human GLUT1, which transports D-glucose independent of proton coupling, was elucidated^19^. Despite the presence of these structures, our understanding of most other glucose transporters still derives largely from modelling studies. Although these homology-based structures provide quite accurate descriptions of transporter topography and helix-packing arrangements, they fail to accurately predict helix and amino acid side chain orientation within and around the active sites. Systematic mutational analyses of these glucose transporters are therefore still necessary for a better understanding of their structure-function relationships.

The filamentous fungus *Trichoderma reesei* (teleomorph *Hypocrea jecorina*) is one of the most prolific industrial cellulase producers due to its excellent capacity to secret large quantities of cellulases. The synergistic actions of the cellulase system can efficiently degrade crystalline cellulose to glucose, the preferred carbon source for *T. reesei*^20^. Although sugar permeases, especially those for lactose, have been identified in *T. reesei* that not only mediate the uptake of the sugar substrate but also participate in the induced production of cellulases by lactose^21^,^22^, the glucose transport system in *T. reesei* is far from being well characterized. In our previous work, we identified the *T. reesei* transporter Stp1, which is capable of supporting the growth of a *S. cerevisiae* strain deficient in all glucose transporters^23^. In this study, Stp1 was characterized in *S. cerevisiae* to better understand its structural features with respect to its sugar transporting activity. Its structure-function relationship was studied by mutational analyses including site-directed mutagenesis and directed protein evolution.

## Results

### Functional characterization of the glucose transporter Stp1 from *T. reesei* in *S. cerevisiae*

**Figure 1:**
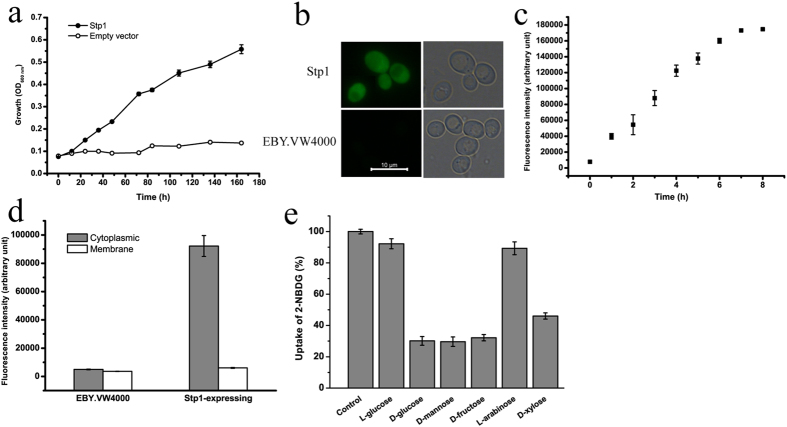
Functional characterization of the glucose transporter Stp1 from the filamentous fungus *T. reesei* in *S. cerevisiae* EBY.VW4000. (**a**) Growth curve of EBY.VW4000 cells expressing Stp1 on 1% glucose. (**b**) Fluorescence microscopic analysis of yeast cells expressing Stp1 after incubation with 2-NBDG for 3 h. (**c**) Time-dependent uptake of 2-NBDG by Stp1-expressing yeast cells. (**d**) Quantitative analysis of the 2-NBDG fluorescent signals of the cytoplasmic and membrane fractions of Stp1-expressing yeast cells after a 3-h incubation with 2-NBDG. The cytoplasmic and membrane fractions of the parental EBY.VW4000 cells were used as a control. (**e**) Substrate competition analysis by measuring the effects of different sugars on 2-NBDG uptake by yeast cells expressing Stp1. Control, control experiment without the addition of sugar competitors; the averaged readout of the control experiments is set as 100%. Values in all panels are the mean of three biological replicates. Error bars are the SD from these replicates.

**Figure 2:**
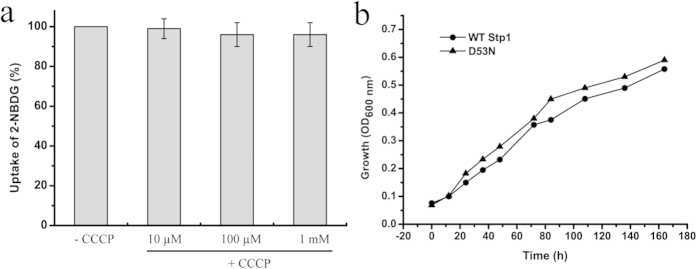
Stp1-mediated 2-NBDG transport is probably independent of proton coupling. (**a**) The effect of CCCP at concentrations from 10–1,000 μM on the uptake of 2-NBDG by yeast cells expressing Stp1. The averaged readout of the control experiments untreated with CCCP is set as 100%. (**b**) The effect of the D53N mutation on the glucose transport capability of Stp1 as measured by the growth of yeast cells in which the variant D53N was expressed. Values in the panels are the mean of three biological replicates. Error bars are the SD from these replicates.

The heterologous expression of *stp1* in *S. cerevisiae* EBY.VW4000, which exhibits deficient growth with hexose due to the lack of hexose transporters^24^, enabled the strain to grow on glucose, demonstrating that Stp1 is capable of transporting glucose. *S. cerevisiae* EBY.VW4000 cells expressing Stp1 displayed a low growth rate (0.016 ± 0.004 h^−1^) on glucose (Fig. 1a), suggesting that Stp1 possesses low glucose transport activity in yeast. We employed 2-NBDG (2-[N-(7-nitrobenz-2-oxa-1,3-diazol-4-yl)amino]-2-deoxy-D-glucose), a fluorescent D-glucose analogue^25^,^26^, to further characterize the transport activity of Stp1. Yeast cells expressing Stp1 were incubated with 2-NBDG at 30 °C, followed by five washes, and the uptake of 2-NBDG was evaluated using fluorescence microscopy and a spectrofluorometer. As shown in Fig. 1b,c, in contrast to parental cells, cells expressing Stp1 were highly fluorescent, indicating that 2-NBDG acts as a ligand for Stp1. To further verify that 2-NBDG was indeed a substrate of Stp1, which is fully capable of catalyzing its transport, the intracellular accumulation of 2-NBDG was quantified by determining the fluorescence intensity of 2-NBDG in the cytoplasmic extracts relative to the cytoplasmic membrane (Fig. 1d). The results showed that, in accordance with what was observed in the microscopic analysis, the vast majority of the 2-NBDG-specific fluorescent signal was largely observed in the cytoplasmic fractions, whereas no significant level of 2-NBDG-specific fluorescence was observed to be associated with the membrane. Substrate competition studies showed that, in contrast to L-glucose and arabinose, sugars including D-glucose, D-mannose, D-fructose, D-galactose and D-xylose could significantly inhibit the uptake of 2-NBDG (Fig. 1e). The inhibitory effect of D-mannose, D-fructose and D-galactose was consistent with the observation that Stp1 expression could also support the growth of EBY.VW4000 cells with the above three sugars as the sole carbon source (Supplementary Fig. S1). To determine the mechanism of substrate transport by Stp1, we measured the effect of carbonyl cyanide m-chlorophenyl hydrazone (CCCP), a protonophore that abolishes Δμ~H+, on the uptake of 2-NBDG by Stp1. In contrast to the well-defined glucose/xylose-H^+^ symporter Gxs1^27^ for which an apparent inhibition of 2-NBDG uptake by CCCP was observed (Supplementary Fig. S2), the addition of CCCP at a final concentration up to 1 mM had almost no effect on 2-NBDG uptake by Stp1 (Fig. 2a). Moreover, mutation of Asp 53, the residue that corresponds to Asp 22 having a critical role in proton coupling in the glucose/H^+^ symporter GlcP^18^, had no negative effect on the glucose-transport capability of Stp1 (Fig. 2b). These results indicated that Stp1 may not be a proton symporter that utilizes the plasma membrane proton gradient.

### Modelled structure of Stp1 and site-directed mutagenesis of the residues putatively participating in glucose recognition

**Figure 3:**
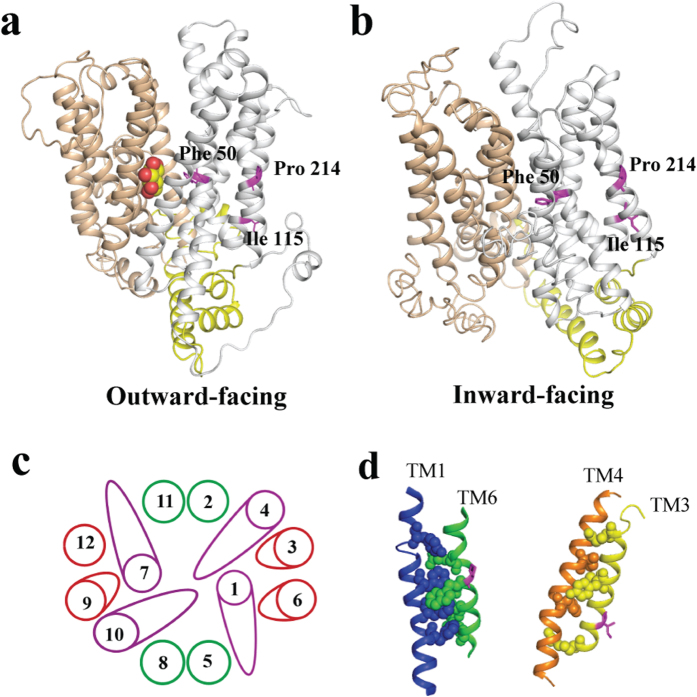
Modelled structures of Stp1 and its putative helix-packing arrangement. Modelled structures were performed with the crystal structures of XylE bound to D-glucose (**a**) and GLUT1 in an inward-open state (**b**) as the template, respectively. The N and C domains are coloured grey and tan, respectively, and intracellular helices connecting these two domains are coloured yellow. D-glucose is indicated by the spheres. (**c**) Putative helix-packing arrangement viewed from the extracellular surface. (**d**) Putative stacking of TMs 6 and 1 (left) and TMs 3 and 4 (right). Residues with putative interactions are shown as spheres. Phe 50, Ile 115 and Pro 214 are represented in magenta.

**Figure 4:**
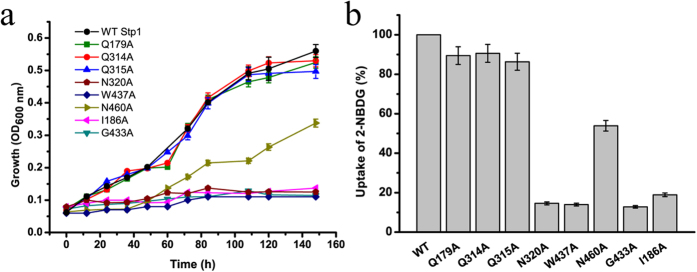
The effects of the individual mutations of several conserved residues putatively participating in substrate binding on the growth-supporting and 2-NBDG-transporting capabilities of Stp1. (**a**) Growth curves of yeast cells expressing the respective variants as indicated on 1% glucose. (**b**) Fluorometric analysis of 2-NBDG uptake by yeast cells expressing the respective variants after a 4-h incubation with 2-NBDG. The averaged readout of the control experiments with WT Stp1 is set as 100%. Values in the panels are the mean of three biological replicates. Error bars are the SD from these replicates.

With the crystal structures of *E. coli* XylE bound to D-glucose and human GLUT1 in an inward-open state as templates, homology modelling of Stp1 was performed. The modelled structure of Stp1 with either template contains a typical major facilitator superfamily fold of 12 transmembrane α-helices and N- and C-terminal domains comprising six-membrane helices (Fig. 3a,b). Sequence alignment demonstrated that the residues hydrogen-bonded to both D-glucose and D-xylose identified in XylE are conserved in glucose transporters, corresponding to Gln 179, Gln 314, Gln 315, Asn 320, Trp 437 and Asn 460 in Stp1 (Supplementary Fig. S3). To examine the role of these conserved residues in glucose transport, several Stp1 variants were generated, each containing the targeted replacement of the specific residues listed above with Ala, and were examined for the resulting transport capabilities using a yeast-based assays. While the Ala substitutions of Gln 179, Gln 314, or Gln 315 had almost no effect on the glucose and 2-NBDG transport activities of Stp1, mutations of Asn 320, Trp 437 or Asn 460 resulted in a drastic effect; N320A and W437A in particular displayed an almost abrogated transport phenotype (Fig. 4), indicating that, unlike the other conserved Gln 179, Gln 314 and Gln 315, these residues play important roles in glucose transport in Stp1. Similarly to N320A and W437A, the targeted Ala substitutions of Ile 186 and Gly 433 in Stp1, two residues that correspond to Gln 175 and Gly 433 in XylE and directly interact with D-glucose but not D-xylose^17^, also nearly abolished the glucose and 2-NBDG transport capability of Stp1 (Fig. 4). Ile 186 and Gly 433 are invariant in GLUTs and homologous glucose transporters (Supplementary Fig. S3), demonstrating important roles of these two residues in glucose transport. Of note, unlike N320A and I186A, the targeted Ala mutations of G433 and W437 probably disrupted the structure of Stp1, as suggested by the microscopic analysis of the cellular localization of the mutants fused with a C-terminal green fluorescence protein (GFP) (Supplementary Fig. S4a). Taken together, these results indicate that residues potentially involved in substrate binding in Stp1 contributed differentially to the transport of glucose.

### Site-directed mutagenesis of Phe 50 proximal to the glucose-binding site

**Figure 5:**
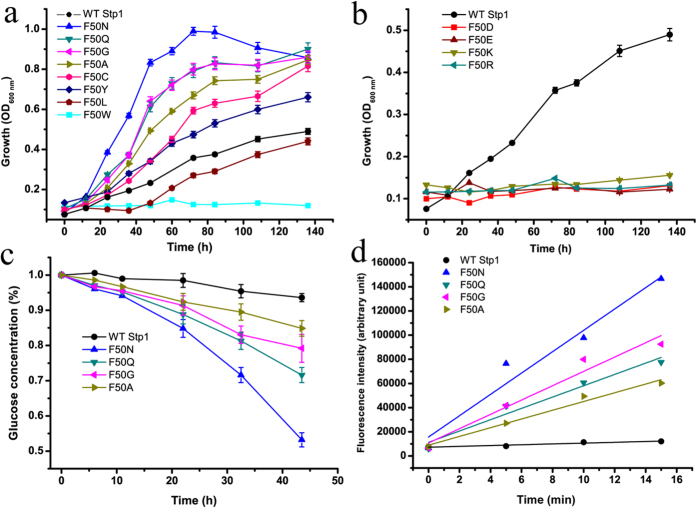
The effect of site-directed mutagenesis of Phe 50 on the glucose- and 2-NBDG-transport capabilities of Stp1. (**a**,**b**) Growth curves of yeast cells expressing the respective variants of Phe 50 as indicated with 1% glucose as the sole carbon source. (**c**) HPLC analysis of the residual glucose in the culture supernatants of the yeast cells expressing the respective variants. (**d**) Fluorometric analysis of 2-NBDG uptake by the respective variants, obtained by measuring the fluorescence intensity of the yeast transformants incubated with 2-NBDG for the indicated time period. Values in all the panels are the mean of three biological replicates.

The presence of aromatic residues surrounding the substrate-binding site is commonly observed in MFS transporters^17^. In addition to Trp 437 described above, Phe 50, Tyr 324 and Trp 461 in Stp1 are highly conserved among glucose transporters and are located in the vicinity of the substrate in XylE^17^ (Supplementary Figs. S3 and S5a). The targeted substitution of Tyr 324 or Trp 461 with Ala led to nearly complete abrogation of glucose transport activity. In contrast, the mutation of Phe 50 to Ala promoted yeast growth on glucose (Supplementary Fig. S5b). We therefore selected Phe 50 for further study. Yeast growth assays demonstrated that replacement of Phe 50 with the relatively hydrophilic aromatic amino acid Tyr^28^ moderately promoted glucose transport activity, whereas replacement with the bulky aromatic residue Trp nearly abolished glucose transport (Fig. 5a). Substitutions with charged residues including Asp, Glu, Lys and Arg resulted in the abrogation of the glucose transport capability of Stp1 (Fig. 5b), which was in accordance with the observation that no charged residue is involved in the substrate-binding site of XylE^17^. The deficiency in transport capability caused by replacement with the charged residues largely derived from compromised catalysis, but not structural disruption because microscopic analysis indicated that the corresponding variants fused with C-terminal GFP were partly located in the membrane periphery of yeast cells (Supplementary Fig. S4a). The replacement of Phe 50 with other polar or non-polar residues exerted differential effects on the activity of Stp1. Expression of the F50L variant substituted with aliphatic leucine slightly retarded yeast growth on glucose, whereas expression of variants substituted with more hydrophilic residues including Cys, Gly, Asn and Gln significantly improved yeast growth (Fig. 5a and Table 1). Specifically, the F50N variant displayed the highest glucose transport activity with a growth rate of 0.055 h^−1^ in comparison with that of WT Stp1 (0.016 h^−1^). In accordance with the significantly promoted growth rate, expression of F50N, F50Q, F50G or F50A led to the more efficient consumption of extracellular glucose versus WT Stp1 when cultured on 1% glucose, indicating the apparently improved glucose transport capabilities of the variants (Fig. 5c). The yeast-based 2-NBDG assay showed that these same variants all displayed an apparently higher uptake rate of 2-NBDG than that of WT Stp1 (Fig. 5d). To determine whether the significantly enhanced glucose and 2-NBDG uptake by these four variants is due to elevated expression levels, we tagged these four mutants with a C-terminal GFP fragment and compared the resulting fluorescence intensity in yeast cells with those expressing WT Stp1. Fluorescence microscopic analysis indicated that these variants were properly targeted to the cell membrane (Supplementary Fig. S4a), and the cells expressing the four variants displayed very similar fluorescence intensities as those expressing WT Stp1 (Supplementary Fig. S4b). These results imply that Phe 50 plays an important role in the transport of glucose, probably by exerting an additional effect on substrate binding or passage.

### Combined mutations of I115F and P214L in Stp1 significantly enhanced glucose transport activity

**Figure 6:**
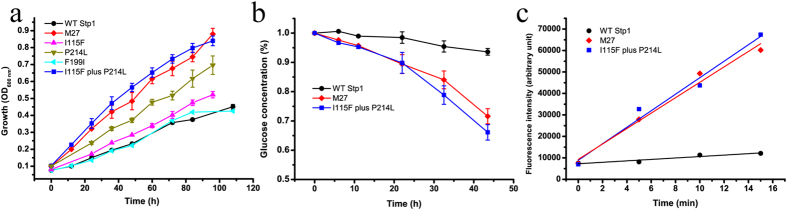
The effects of the individual or combined mutations contained in variant M27 on the glucose and 2-NBDG transport capabilities of Stp1. (**a**) Growth curves of yeast cells expressing the indicated variants with 1% glucose as the sole carbon source. (**b**) HPLC analysis of the residual glucose in the culture supernatants of the yeast cells expressing the respective variants. (**c**) Fluorometric analysis of 2-NBDG uptake by the respective Stp1 variants, obtained by measuring the fluorescence intensity of yeast transformants incubated with 2-NBDG for the indicated time period. Values in all the panels are the mean of three biological replicates.

Directed evolution is an effective method to study the structure-function relationships of enzymes^29^,^30^. We thus performed error-prone PCR to generate a library of Stp1 variants, transformed yeast EBY.VW4000 cells with these variants and screened for colonies with faster growth rates on a plate containing glucose as the sole carbon source. From almost 400 transformants, we obtained a colony carrying a Stp1 variant, named M27, that displayed remarkably faster growth on glucose compared with WT Stp1. DNA sequencing identified that variant M27 differed from WT Stp1 by three amino acid substitutions, I115F, F199I and P214L, while no changes occurred in the other regions of the expression vector. Retransformation confirmed that M27 led to much faster growth on glucose, with a specific growth rate up to 0.040 h^−1^ (Fig. 6a and Table 1). HPLC analysis of the extracellular residual glucose further revealed that yeast cells expressing M27 consumed glucose at a much faster rate compared with WT Stp1-expressing cells (Fig. 6b). In accordance with the yeast growth rate measurements, variant M27 displayed an improved 2-NBDG uptake rate relative to that of WT Stp1 (Fig. 6c). The higher transport activity exhibited by M27 was not due to a higher expression level (Supplementary Fig. S4). To further distinguish the contribution of each mutation to the enhanced activity of M27, we constructed variants with the individual mutations I115F, F199I or P214L using site-directed mutagenesis. While the expression of F199I led to a similar growth rate as that of WT Stp1, expression of either variant I115F or P214L improved yeast growth on glucose (P214L > I115F), although the growth rates were lower than that of M27 (Fig. 6a and Table 1). Combining I115F and P214L mutations resulted in similar growth performance, glucose consumption and 2-NBDG uptake in yeast cells as those expressing variant M27 (Fig. 6 and Table 1), indicating that the combination of these two mutations fully accounted for the enhanced specific glucose and 2-NBDG transport activities in M27. Analysis of the modelled structure of Stp1 revealed that, unlike Phe 50, which is positioned proximal to the substrate-binding site, the non-conserved residues Ile 115 and Pro 214 are located on the lateral surface of the N-domain, with Ile 115 on TM 3 and Pro 214 on TM 6 (Fig. 3).

## Discussion

Glucose transport is the first step, as well as the rate-limiting step, towards glucose utilization. Despite the availability of genomic information on diverse fungi, including the model cellulolytic fungus *T. reesei*, a comprehensive study of their sugar transporters has been lacking. In this study, we characterized the glucose transporter Stp1 from *T. reesei* in the hexose transporter-null yeast strain EBY.VW4000. The ability of Stp1 and its variants to restore the yeast growth was used to evaluate their transport activity for glucose. We also employed a fluorescent derivative of D-glucose, 2-NBDG, to further characterize the transport activity of Stp1. The quantitative analysis of the cellular distribution of 2-NBDG-specific fluorescence verified that 2-NBDG is a substrate of Stp1, which is able to catalyze its intracellular transport. The uptake of 2-NBDG was also inhibited by glucose in a dose-dependent manner (Supplementary Fig. S6). Importantly, the Stp1 variants whose expression significantly promoted yeast growth on not only glucose but also other sugar substrates (Supplementary Fig. S1) took up 2-NBDG at a much higher rate; the cytoplasmic 2-NBDG-specific signal appeared after only minutes (Supplementary Fig. S7). Taken together, these results indicated that 2-NBDG in conjunction with yeast strain EBY.VW4000 could be reasonably used to probe the structural determinants of Stp1 glucose transport.

To explore the structural and functional features of Stp1, site-directed mutagenesis was first performed using the available structures of XylE and GLUT1, both of which are typical MFS sugar transporters. Six residues including Gln 179, Gln 314, Gln 315, Asn 320, Trp 437 and Asn 460 from Stp1, all of which are within hydrogen-bonding range of the substrate as revealed by the crystal structure of XylE, are highly conserved in GLUTs and their homologues. In XylE, individual mutation of these residues significantly compromised xylose transport activity^17^. Similarly, the corresponding residues in GLUT1 are critical for glucose transport^31^. The observation that only three (Asn 320, Trp 437 and Asn 460) of the six residues in Stp1 were critical for the transport for glucose implies that these conserved amino acids probably make different contributions to the binding of glucose in different glucose transporters and that Stp1 may adopt a somewhat different substrate-binding mode from that of XylE and GLUT1 during transport. The mutation of Ile 186 or Gly 433, amino acids corresponding to the two extra residues that hydrogen-bond D-glucose but not D-xylose in *E. coli* XylE, almost completely abolished the transporting activity of Stp1. A sequence alignment showed that the corresponding sites of Ile 186 and Gly 433 are highly conserved in GLUTs, suggesting that these two residues play important roles in glucose transport. Due to the lack of a crystal structure of GLUT1 bound to D-glucose, it is unclear whether these two conserved sites are directly involved in glucose binding. However, microscopic analysis of the cellular localization of the two variants indicated that, unlike I186A, the mutation G433A completely disrupted the structure of Stp1. It is thus tempting to speculate that Gly 433 in TM10 is critical for maintaining structural stability but is not involved in substrate binding.

Phe 50 is an invariant residue located in the vicinity of the glucose-binding site (Fig. 3 and Supplementary Fig. S3), and substitution of the corresponding site in XylE (Phe 24) with Ala significantly compromised its xylose transport activity^17^. Unlike the other three aromatic residues proximal to the substrate (Trp 437, Phe 324 and Trp 461), substitution of Phe 50 with Asn, Gln, Gly or Ala markedly improved the glucose- and 2-NBDG-transport capabilities of Stp1; the variant F50N displayed the strongest ability to support yeast growth. Together with the observation that targeted changes of Phe 50 to charged residues or the more bulky tryptophan almost abolished the ability of Stp1 to support yeast growth on glucose, it is assumed that the amino acid at this site has a profound influence on the transport process by either facilitating the access of glucose into the binding pocket or contributing to the inward release of glucose during the alternating conformational changes of the transporter.

By using directed protein evolution with error-prone PCR, we also obtained the Stp1 variant M27, which exhibits significantly enhanced glucose transport activity and contains the three mutations I115F, F199I and P214L. Further analysis demonstrated that the enhanced glucose transport capability of M27 could be fully attributed to the combined mutations of I115F and P214L. Unlike Phe 50, which is positioned around the substrate-binding site, Ile 115 and Pro 214 are located on the lateral surface of the Stp1 N-domain on TM3 and TM6, respectively. The modelled structure revealed that TM3 and TM6 scaffolds are juxtaposed with the translocation pore-forming TM1 and TM4 scaffolds (Fig. 3c). It is therefore very likely that extensive interactions exist between these transmembrane subdomains (Fig. 3c). In this respect, it has been reported that TM6 of GLUT1, although not directly involved in binding the substrate, is both necessary and sufficient for trans-acceleration by coordinating membrane-spanning amphipathic helices that form the sugar translocation pore^32^. GLUT1 TM6 has thus been predicted to stabilize the endo- and exofacial orientations of the substrate-deficient carrier, thereby restraining conformational changes between the exo- and endofacial states (e.g., relaxation) to exert an inhibitory effect on sugar uptake^32^. A similar scenario might exist for the M27 variant. One assumption for the observed enhanced activity of M27 is that the combined mutations of I115F and P214L on TM3 and TM6, respectively, somehow alleviate the restraints inflicted on the relative movements of N- and C-domains by modulating TM arrangements and accelerating conformational changes when catalyzing the transport cycle. Considering that Ile 115 and Pro 214 are not conserved in GLUTs or other glucose transporters, careful inspection of the local structural interactions combined with systematic mutational analyses will undoubtedly provide more insights into the structural and mechanistic details of sugar delivery across membranes by Stp1 and other specific transporters. However, the identified mutants represent a starting point to tease apart the potentially repressive effect exerted by Stp1 relative to its glucose transport activity.

## Methods

### Strains, media and cultivation conditions

*S. cerevisiae* EBY.VW4000^24^ cells were routinely cultivated at 30 °C in YPM medium (10 g/litre yeast extract, 20 g/litre peptone and 10 g/litre maltose). For transformant selection, yeast synthetic complete (SC) medium (6.7 g/litre yeast nitrogen base without amino acids, 1.7 g/litre drop-out mix) plus 10 g/litre maltose was used. Uracil or leucine was omitted for plasmid selection. For routine plasmid construction and amplification, *E. coli* DH5α was grown in Luria-Bertani medium with the addition of 100 μg/ml ampicillin as required.

### DNA manipulation

To generate *stp1* fragments with specific site-directed mutations, overlap-extension PCR^33^ was performed with pRS426ADH*stp1*^23^ as the original template. After digestion with EcoRI and HindIII, the mutated *stp1* fragments were ligated into pRS426ADH^23^, which was derived from pRS426 containing the *S. cerevisiae* ADH-1 promoter and terminator. DNA sequencing was performed to confirm that each mutagenesis occurred as expected. To determine the subcellular localization of the Stp1 variants, the coding sequence of the respective variant was fused with that of a green fluorescence protein (GFP) by overlap-extension PCR and subsequently inserted into the EcoRI and HindIII sites of pRS426ADH.

Error-prone PCR was used to create a library of *stp1* mutants. The error-prone PCR reaction mixture contained 1X PCR buffer with MgCl_2_, 0.2 M of each primer, 7 mM MgCl_2_, 0.3 mM MnCl_2_, 0.2 mM dGTP, 0.2 mM dATP, 1 mM dCTP, 1 mM dTTP, 10 ng pRS426ADH*stp1* plasmid DNA as template, and 2.5 units (U) of Taq polymerase. The PCR conditions were as follows: 95 °C for 5 min followed by 30 cycles of 95 °C for 30 s, 55 °C for 30 s and 72 °C for 2 min and a final extension at 72 °C for 10 min. The mutation rate was approximately 3 bp per kb, which was confirmed by DNA sequencing. After digestion with EcoRI and HindIII, the amplification products were incorporated into pRS426ADH.

### Yeast transformation and plasmid isolation

The respective constructs were used to transform yeast strains by the PEG/LiAc method of Gietz *et al.*^34^. For the mutant library generated by error-prone PCR, the Yeastmaker™ Yeast Transformation System 2 kit (Clontech) was used according to the manufacturer’s instructions. Transformants were selected on SC medium containing 10 g/litre maltose. For the screening of colonies for fast growth on glucose, transformants from the maltose plate were streaked on a glucose plate and incubated at 30 °C for 8 days. Plasmid isolation from the yeast cells was performed using the E.Z.N.A.^®^ Yeast Plasmid kit (Omega). The isolated plasmids were amplified through *E. coli* transformation and then subjected to DNA sequencing.

### Yeast growth assay

Transformants were grown in liquid SC medium with 10 g/litre maltose under selective conditions, harvested during logarithmic growth, washed, adjusted to the same cell density and transferred to medium with 10 g/litre glucose, mannose, fructose or galactose as the sole carbon source. The growth curve was determined by measuring the optical density at 600 nm using a 96-well UV-visible spectrophotometer and reported as the mean of three independent experiments obtained from three transformants.

### 2-NBDG assay

Yeast cells expressing Stp1 or its variants were grown on SC media containing maltose to logarithmic growth, washed, and resuspended to a final volume of 200 μl with a 50 mM phosphate buffer at pH 5.6 containing 150 mM NaCl, 5 mM KCl, 2 mM CaCl_2_, and 1 mM MgSO_4_ at a cell density of 2.0 at 600 nm. After the addition of a fluorescent derivative of D-glucose, 2-[N-(7-nitrobenz-2-oxa-1,3-diazol-4-yl)amino]-2-deoxy-D-glucose (2-NBDG) (Life Technologies), at a final concentration of 200 μM, cells were incubated at 30 °C for the indicated time period. After washing with ice-cold phosphate buffer three to five times by centrifugation, the yeast cells were resuspended to a final volume of 200 μl in the same phosphate buffer and analyzed using fluorescence microscopy and a spectrofluorometer. To verify 2-NBDG was intracellularly transported by Stp1, yeast cell fractionation was performed as follows: the yeast cells incubated with 2-NBDG for the indicated time periods were washed and resuspended in phosphate buffer containing 1 mM EDTA and 1 mM PMSF (phenylmethanesulphonyl fluoride). The cells were disrupted by glass beads in a bead beater three times and centrifuged for 20 min at 8,000 × *g*. The supernatant was then centrifuged for 40 min at 300,000 × *g*; the resulting supernatant was separated, and the pellet was resuspended to form the crude membrane fraction. The cytoplasmic supernatant and the membrane suspensions were subsequently subjected to fluorometric analysis. For the substrate competition assay, yeast cells expressing Stp1 were incubated with 100 μM 2-NBDG for 4 h in the presence of a 10-fold molar excess of L-glucose, D-glucose, D-mannose, D-fructose, D-galactose, D-xylose or L-arabinose. To measure the effect of carbonyl cyanide m-chlorophenyl hydrazone (CCCP) on 2-NBDG uptake by Stp1, CCCP at final concentrations from 10–1,000 μM was added to the Stp1-expressing yeast cells 5 min prior to the addition of 2-NBDG. The incubation was continued at 30 °C for 30 min and stopped by washes with the ice-cold phosphate buffer. Cells were resuspended to a final volume of 200 μl in the same phosphate buffer and subjected to fluorometric analysis.

### Fluorometric analysis

The fluorescence of yeast cells expressing the Stp1 variants fused to C-terminal GFP or incubated with 2-NBDG was detected using a Nikon Eclipse 80i fluorescence microscope. For the quantification of fluorescence intensity from GFP or 2-NBDG, the yeast cells or the cellular fractions were transferred to a black microtitre plate and measured using a 96-well spectrofluorometer at an excitation wavelength of 485 nm and an emission wavelength of 535 nm. An aliquot of each sample was diluted for OD_600_ determination to calculate the relative fluorescence for each sample per OD.

### HPLC analysis

The analysis of media glucose content by HPLC (high performance liquid chromatography) was performed as follows: after filtration with a 0.22-μm membrane, medium supernatants were applied to a Bio-Rad Aminex HPX-42A carbohydrate column and analyzed with an LC-10AD HPLC (Shimadzu, Japan) equipped with a RID-10A refractive index detector. The column was maintained at 78 °C and eluted with double-distilled water at a flow rate of 0.4 ml/min.

### Homology modelling

The three-dimensional model of Stp1 was constructed using the I-TASSER protein modelling server^35^ with the crystal structures of XylE (PDB NO. 4GBZ) and GLUT1 (PDB NO. 4PYP) as templates. I-TASSER builds 3D models through an exhaustive process that involves automatic template selection, fragment reassembly of aligned regions, ab initio modelling of unaligned regions, clustering, energy evaluation and the optimization of a model’s hydrogen-bonding network. The model quality evaluation automatically calculated by I-TASSER indicated that the resulting model had a high confidence (C-score = −0.78, Estimated TM Score = 0.61 ± 0.14). Visualization and analysis of the modelled structure was performed with PyMOL.

## Additional Information

**How to cite this article**: Zhang, W. *et al.* Identification of residues important for substrate uptake in a glucose transporter from the filamentous fungus *Trichoderma reesei*. *Sci. Rep.* **5**, 13829; doi: 10.1038/srep13829 (2015).

## Supplementary Material

Supplementary Information

## Figures and Tables

**Table 1:** The maximum growth rates of yeast cells expressing WT Stp1 or its variants.

WT and Variant	Growth rate (h^−1^)
WT	0.016 ± 0.004
F50N	0.055 ± 0.001
F50Q	0.037 ± 0.000
F50G	0.041 ± 0.001
F50A	0.035 ± 0.002
F50C	0.025 ± 0.001
F50Y	0.022 ± 0.002
M27	0.040 ± 0.001
I115F	0.024 ± 0.004
P214L	0.033 ± 0.000
I115F plus P214L	0.041 ± 0.002

## References

[b1] Thorens B. & Mueckler M. Glucose transporters in the 21st Century. Am J Physiol Endocrinol Metab 298, E141–145 (2010). 20009031 10.1152/ajpendo.00712.2009 PMC2822486

[b2] Herman M. A. & Kahn B. B. Glucose transport and sensing in the maintenance of glucose homeostasis and metabolic harmony. J Clin Invest 116, 1767–1775 (2006). 16823474 10.1172/JCI29027 PMC1483149

[b3] Scheepers A., Joost H. G. & Schurmann A. The glucose transporter families SGLT and GLUT: molecular basis of normal and aberrant function. JPEN J Parenter Enteral Nutr 28, 364–371 (2004). 15449578 10.1177/0148607104028005364

[b4] Mueckler M. & Thorens B. The SLC2 (GLUT) family of membrane transporters. Mol Aspects Med 34, 121–138 (2013). 23506862 10.1016/j.mam.2012.07.001 PMC4104978

[b5] Ozcan S. & Johnston M. Function and regulation of yeast hexose transporters. Microbiol Mol Biol Rev 63, 554–569 (1999). 10477308 10.1128/mmbr.63.3.554-569.1999 PMC103746

[b6] Ozcan S. & Johnston M. Three different regulatory mechanisms enable yeast hexose transporter (HXT) genes to be induced by different levels of glucose. Mol Cell Biol 15, 1564–1572 (1995). 10.1128/mcb.15.3.1564 PMC230380 7862149

[b7] Maier A., Volker B., Boles E. & Fuhrmann G. F. Characterisation of glucose transport in Saccharomyces cerevisiae with plasma membrane vesicles (countertransport) and intact cells (initial uptake) with single Hxt1, Hxt2, Hxt3, Hxt4, Hxt6, Hxt7 or Gal2 transporters. FEMS Yeast Res 2, 539–550 (2002). 10.1111/j.1567-1364.2002.tb00121.x 12702270

[b8] Vankuyk P. A. *et al.* Aspergillus niger mstA encodes a high-affinity sugar/H+ symporter which is regulated in response to extracellular pH. Biochem J 379, 375–383 (2004). 14717659 10.1042/BJ20030624 PMC1224080

[b9] Forment J. V. *et al.* High-affinity glucose transport in aspergillus nidulans is mediated by the products of two related but differentially expressed genes. PLoS One 9, e94662 (2014). 24751997 10.1371/journal.pone.0094662 PMC3994029

[b10] Wei H. *et al.* A putative high affinity hexose transporter, hxtA, of Aspergillus nidulans is induced in vegetative hyphae upon starvation and in ascogenous hyphae during cleistothecium formation. Fungal Genet Biol 41, 148–156 (2004). 10.1016/j.fgb.2003.10.006 14732261

[b11] Forment J. V., Flipphi M., Ramon D., Ventura L. & Maccabe A. P. Identification of the mstE gene encoding a glucose-inducible, low affinity glucose transporter in Aspergillus nidulans. J Biol Chem 281, 8339–8346 (2006). 16418173 10.1074/jbc.M508198200

[b12] Dos Reis T. F. *et al.* Identification of glucose transporters in Aspergillus nidulans. PLoS One 8, e81412 (2013). 24282591 10.1371/journal.pone.0081412 PMC3839997

[b13] Pao S. S., Paulsen I. T. & Saier M. H. Jr. Major facilitator superfamily. Microbiol Mol Biol Rev 62, 1–34 (1998). 9529885 10.1128/mmbr.62.1.1-34.1998 PMC98904

[b14] Saier M. H. Jr., Reddy V. S., Tamang D. G. & Vastermark A. The transporter classification database. Nucleic Acids Res 42, D251–258 (2014). 24225317 10.1093/nar/gkt1097 PMC3964967

[b15] Smirnova I., Kasho V. & Kaback H. R. Lactose permease and the alternating access mechanism. Biochemistry 50, 9684–9693 (2011). 21995338 10.1021/bi2014294 PMC3210931

[b16] Abramson J. *et al.* Structure and mechanism of the lactose permease of Escherichia coli. Science 301, 610–615 (2003). 12893935 10.1126/science.1088196

[b17] Sun L. *et al.* Crystal structure of a bacterial homologue of glucose transporters GLUT1-4. Nature 490, 361–366 (2012). 10.1038/nature11524 23075985

[b18] Iancu C. V., Zamoon J., Woo S. B., Aleshin A. & Choe J. Y. Crystal structure of a glucose/H+ symporter and its mechanism of action. Proc Natl Acad Sci USA 110, 17862–17867 (2013). 24127585 10.1073/pnas.1311485110 PMC3816430

[b19] Deng D. *et al.* Crystal structure of the human glucose transporter GLUT1. Nature 510, 121–125 (2014). 24847886 10.1038/nature13306

[b20] Kubicek C. P. The cellulase proteins of Trichoderma reesei: structure, multiplicity, mode of action and regulation of formation Adv. Biochem. Eng.-Biotechnol. 45, 1–27 (1992).

[b21] Ivanova C., Baath J. A., Seiboth B. & Kubicek C. P. Systems Analysis of Lactose Metabolism in Trichoderma reesei Identifies a Lactose Permease That Is Essential for Cellulase Induction. PLoS One 8, e62631 (2013). 23690947 10.1371/journal.pone.0062631 PMC3648571

[b22] Porciuncula Jde O. *et al.* Identification of Major Facilitator Transporters Involved in Cellulase Production during Lactose Culture of Trichoderma reesei PC-3-7. Biosci Biotechnol Biochem 77, 1014–1022 (2013). 23649266 10.1271/bbb.120992

[b23] Zhang W. *et al.* Two major facilitator superfamily sugar transporters from Trichoderma reesei and their roles in induction of cellulase biosynthesis. J Biol Chem 288, 32861–32872 (2013). 24085297 10.1074/jbc.M113.505826 PMC3829138

[b24] Wieczorke R. *et al.* Concurrent knock-out of at least 20 transporter genes is required to block uptake of hexoses in Saccharomyces cerevisiae. FEBS Lett 464, 123–128 (1999). 10618490 10.1016/s0014-5793(99)01698-1

[b25] Yamada K. *et al.* Measurement of glucose uptake and intracellular calcium concentration in single, living pancreatic beta-cells. J Biol Chem 275, 22278–22283 (2000). 10748091 10.1074/jbc.M908048199

[b26] Blodgett A. B. *et al.* A fluorescence method for measurement of glucose transport in kidney cells. Diabetes Technol Ther 13, 743–751 (2011). 21510766 10.1089/dia.2011.0041 PMC3118926

[b27] Leandro M. J., Goncalves P. & Spencer-Martins I. Two glucose/xylose transporter genes from the yeast Candida intermedia: first molecular characterization of a yeast xylose-H+ symporter. Biochem J 395, 543–9 (2006). 16402921 10.1042/BJ20051465 PMC1462686

[b28] Kyte J. & Doolittle R. F. A simple method for displaying the hydropathic character of a protein. J Mol Biol 157, 105–132 (1982). 7108955 10.1016/0022-2836(82)90515-0

[b29] Eriksen D. T., Hsieh P. C., Lynn P. & Zhao H. Directed evolution of a cellobiose utilization pathway in Saccharomyces cerevisiae by simultaneously engineering multiple proteins. Microb Cell Fact 12, 61 (2013). 23802545 10.1186/1475-2859-12-61 PMC3702475

[b30] Lian J., Li Y., HamediRad M. & Zhao H. Directed evolution of a cellodextrin transporter for improved biofuel production under anaerobic conditions in Saccharomyces cerevisiae. Biotechnol Bioeng 111, 1521–1531 (2014). 24519319 10.1002/bit.25214

[b31] Olsowski A., Monden I., Krause G. & Keller K. Cysteine scanning mutagenesis of helices 2 and 7 in GLUT1 identifies an exofacial cleft in both transmembrane segments. Biochemistry 39, 2469–2474 (2000). 10704196 10.1021/bi992160x

[b32] Vollers S. S. & Carruthers A. Sequence determinants of GLUT1-mediated accelerated-exchange transport: analysis by homology-scanning mutagenesis. J Biol Chem 287, 42533–42544 (2012). 23093404 10.1074/jbc.M112.369587 PMC3522255

[b33] Heckman K. L. & Pease L. R. Gene splicing and mutagenesis by PCR-driven overlap extension. Nat Protoc 2, 924–932 (2007). 17446874 10.1038/nprot.2007.132

[b34] Gietz D. St Jean A., Woods R. A. & Schiestl R. H. Improved method for high efficiency transformation of intact yeast cells. Nucleic Acids Res 20, 1425 (1992). 1561104 10.1093/nar/20.6.1425 PMC312198

[b35] Roy A., Kucukural A. & Zhang Y. I-TASSER: a unified platform for automated protein structure and function prediction. Nat Protoc 5, 725–738 (2010). 20360767 10.1038/nprot.2010.5 PMC2849174

