# Supplementary material for: Identification of residues important for substrate uptake in a glucose transporter from the filamentous fungus Trichoderma reesei
Source: Sci Rep. 2015 Sep 8;5:13829. doi: 10.1038/srep13829 (PMC4642563; doi:10.1038/srep13829)
Supplement: Supplementary Information [file srep13829-s1.pdf]

Identification of residues important for substrate uptake in a glucose transporter from the filamentous fungus *Trichoderma reesei*

Weixin Zhang[1,+], Yanli Cao[1,+], Jing Gong[2], Xiaoming Bao[1], Guanjun Chen[1], Weifeng Liu[1]*

[1]State Key Laboratory of Microbial Technology, School of Life Science, Shandong University, No.27 Shanda South Road, Jinan 250100, Shandong, P. R. China

[2]Cancer Research Center, School of Medicine, Shandong University, No.44 Wenhuaxi Road, Jinan 250012, Shandong, P. R. China

[+]These two authors contributed equally to this work.

*Correspondence should be addressed to Weifeng Liu. Tel.:+86 531 88364324; Fax: +86 531 88565610; e-mail: weifliu@sdu.edu.cn

Supplementary data

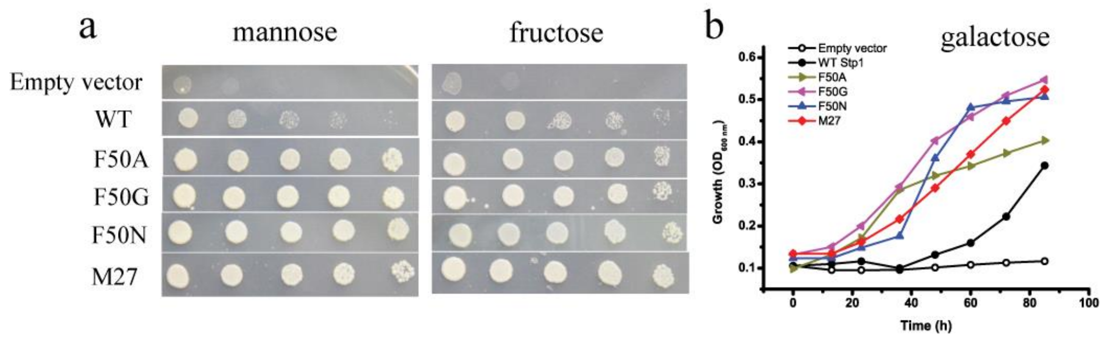

Figure S1. Growth analysis of *S. cerevisiae* EBY.VW4000 cells expressing Stp1 or its variants with mannose, fructose or galactose as the sole carbon source. (a) Serial dilutions of yeast cells were spotted on a synthetic complete agar plate for 5 days with 1% mannose or 1% fructose as the sole carbon source. (b) Growth curves of yeast cells cultured on 1% galactose for the indicated time period. *S. cerevisiae* EBY.VW4000 cells displayed moderate background growth on the agar plate containing 1% galactose; growth in liquid medium was thus measured instead of that on plates.

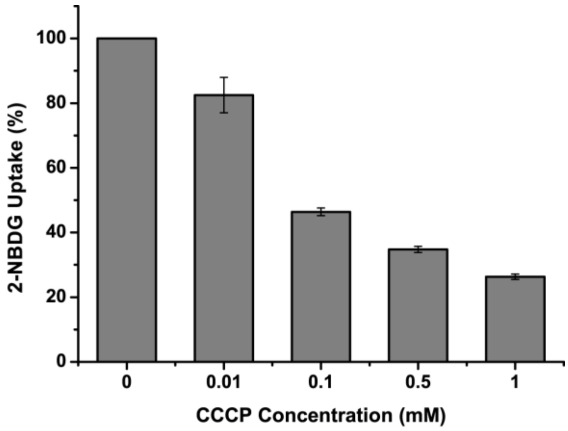

Figure S2 The effect of CCCP on the 2-NBDG uptake by EBY.VW4000 cells expressing Gxs1, a well-defined glucose/xylose-H^+ symporter from *Candida intermedia*. The *gxs1* expression cassette that contains TDH3 promoter, *gxs1* ORF and CYC1 terminator in order was integrated into the transformant genome. Yeast transformant expressing Gxs1 was cultured in SC medium with 1% maltose and 250 μg/ml G418. The CCCP assay was performed under the same experimental conditions as that on Stp1. CCCP at the indicated final concentrations was added to Stp1-expressing yeast cells 5 min prior to the addition of 2-NBDG, and then the incubation was continued at 30 ℃ for 30 min and stopped by three washes with ice-cold buffer.

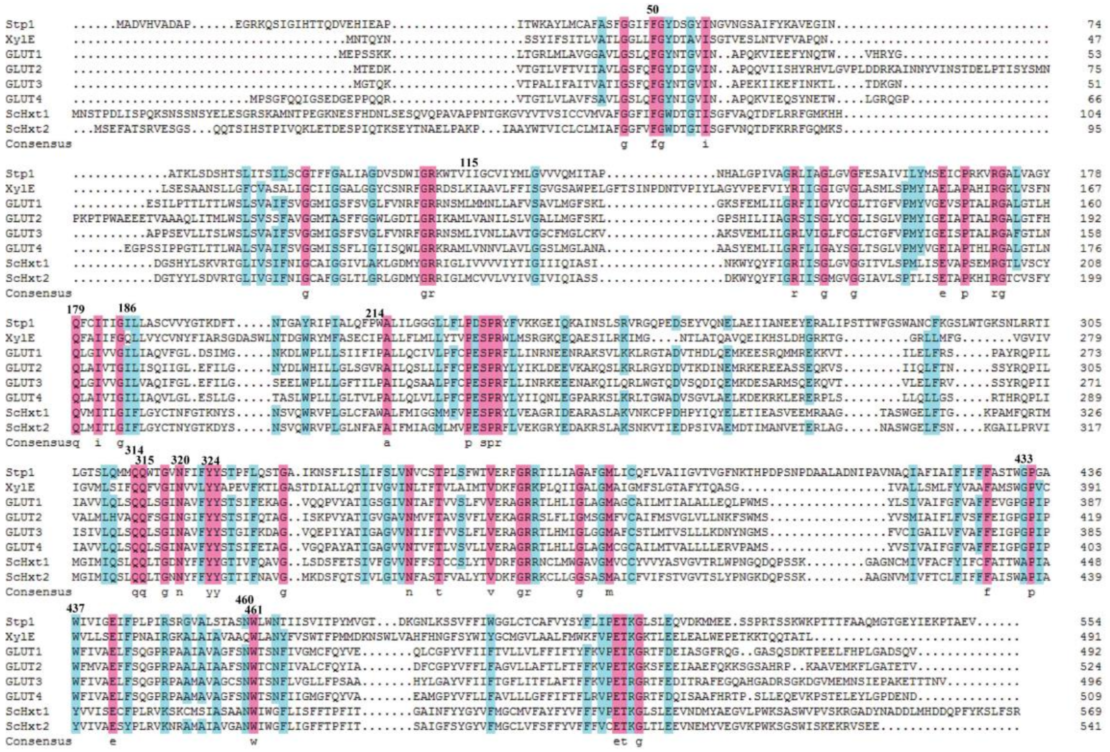

Figure S3 Sequence alignment of Stp1 with its homologues including the xylose/H$^+$ symporter XylE from *E. coli*, human GLUT1-GLUT4, and Hxt1 and Hxt2 from *S. cerevisiae.* The multiple sequence alignments was performed with ClustalW, and the result was constructed with DNAMAN. The residues in Stp1 described in this study are indicated with their corresponding residue numbers.

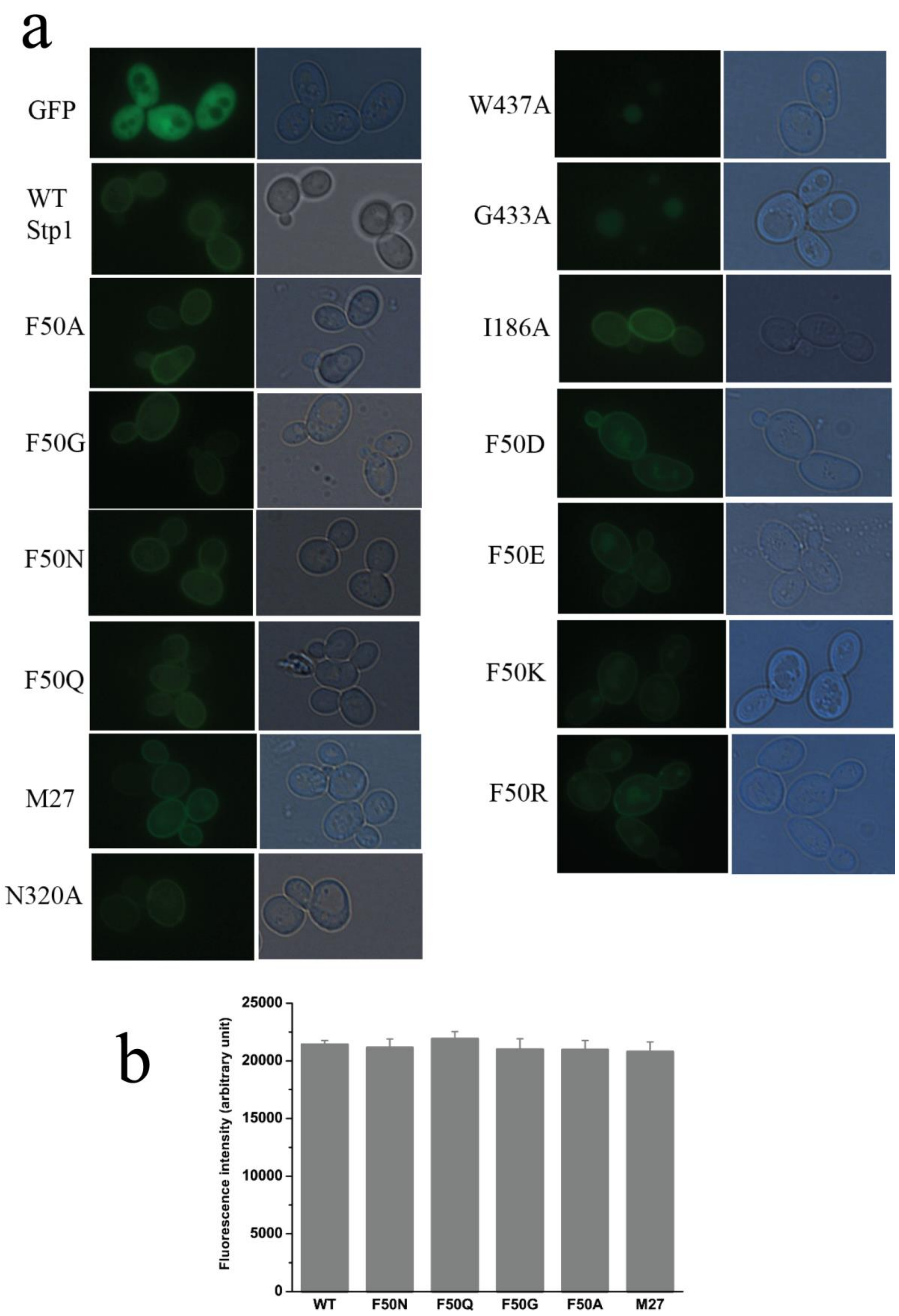

Figure S4 (a) Cellular localization of Stp1 variants illustrated by fluorescence microscopic analysis of EBY.VW4000 yeast cells

expressing the respective variants fused with C-terminal GFP. Yeast cells expressing GFP alone were used as the control, in which fluorescence of GFP was distributed in the cytoplasmic space. (b) Analysis of fluorescence intensity of yeast cells expressing WT Stp1 and the indicated variants fused with C-terminal GFP. The yeast cells cultured in synthetic complete medium containing 1% maltose as the sole carbon source were collected, resuspended in a 25-mM phosphate buffer at pH 5.2 and subjected to fluorescence measurement.

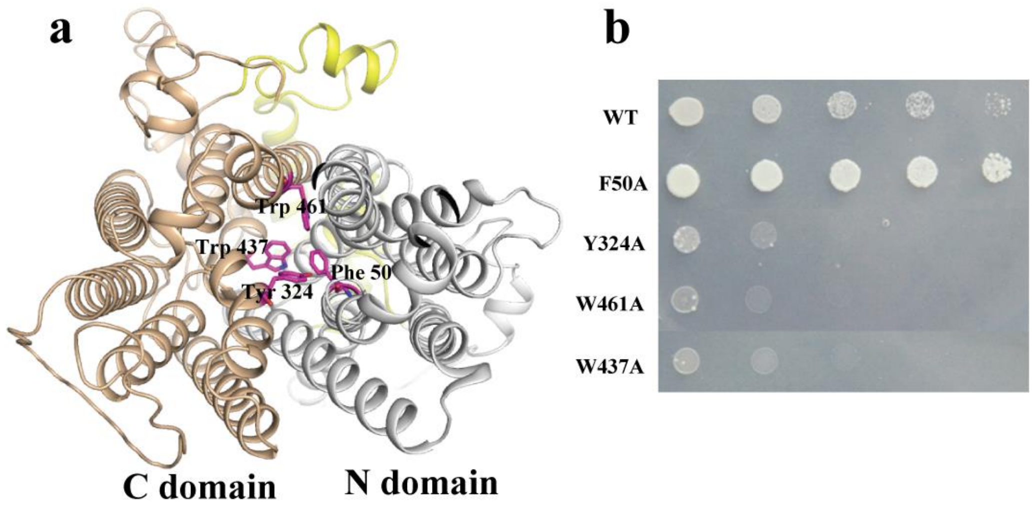

Figure S5 Individual mutation of the aromatic residues including Tyr 324, Trp 461 and Trp 437 in the vicinity of the substrate-binding site of Stp1 nearly abolished its glucose transport capability. (a) Top view of the modelled structure of Stp1 with the crystal structure of *E. coli* XylE as template. The N and C domains are coloured grey and tan, respectively, and the intracellular helices connecting these two domains are coloured yellow. Phe 50, Tyr 324, Trp 461 and Trp 437 are highlighted in magenta. (b) Glucose-mediated growth of *S. cerevisiae* EBY.VW4000 expressing Stp1 and its variants as indicated. Serial dilutions of the yeast cells were spotted on a synthetic complete agar plate for 8 days with 1% glucose as the sole carbon source.

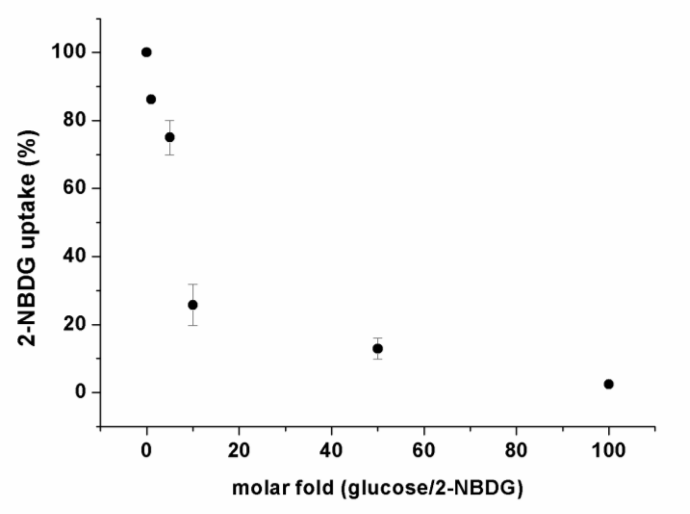

Figure S6 The effect of dose-dependent inhibition by glucose on 2-NBDG uptake by Stp1. Yeast cells expressing Stp1 were incubated with 100 μM 2-NBDG plus excess glucose as indicated for 4 h and then subjected to fluorometric analysis after washing with ice-cold phosphate buffer.

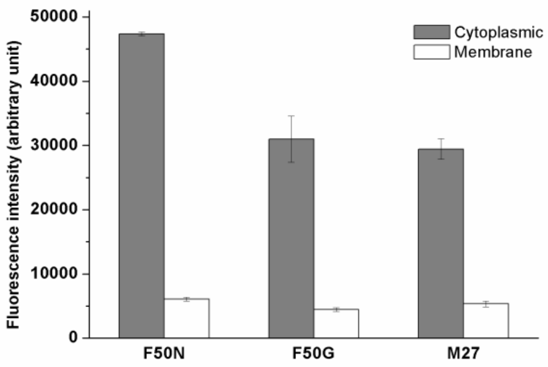

Figure S7 Quantitative analysis of the 2-NBDG-specific fluorescent signals in the cytoplasmic and membrane fractions of yeast cells expressing the variants as indicated after a 5-min incubation with 2-NBDG.
